# Env-Specific Antibodies in Chronic Infection versus in Vaccination

**DOI:** 10.3389/fimmu.2017.01057

**Published:** 2017-09-04

**Authors:** Martina Soldemo, Gunilla B. Karlsson Hedestam

**Affiliations:** ^1^Department of Microbiology, Tumor and Cell Biology, Karolinska Institutet, Stockholm, Sweden

**Keywords:** B cells, HIV-1, neutralizing antibodies, vaccine, HIV-1 Infection

## Abstract

Antibodies are central in vaccine-mediated protection. For HIV-1, a pathogen that displays extreme antigenic variability, B cell responses against conserved determinants of the envelope glycoproteins (Env) are likely required to achieve broadly protective vaccine-induced responses. To understand antibodies in chronic infection, where broad serum neutralizing activity is observed in a subset of individuals, monoclonal antibodies mediating this activity have been isolated. Studies of their maturation pathways reveal that years of co-evolution between the virus and the adaptive immune response are required for such responses to arise. Furthermore, they do so in subjects who display alterations of their B cell subsets caused by the chronic infection, conditions that are distinctly different from those in healthy hosts. So far, broadly neutralizing antibody responses were not induced by vaccination in primates or small animals with natural B cell repertoires. An increased focus on the development vaccine-induced responses in healthy subjects is therefore needed to delineate how the immune system recognizes different forms of HIV-1 Env and to optimize approaches to stimulate antibody responses against relevant neutralizing antibody epitopes. In this review, we describe aspects of Env-directed antibody responses that differ between chronic HIV-1 infection and subunit vaccination for an increased appreciation of these differences; and we highlight the need for an improved understanding of vaccine-induced B cell responses to complex glycoproteins such as Env, in healthy subjects.

## B Cell Subsets in Normal Physiology

The human adaptive immune system relies on several B-lymphocyte subsets with distinct roles. Circulating B cells can be classified as antigen-inexperienced or antigen-experienced cells. Among the former are the immature, transitional B cells and the mature naive B cells. Human transitional B cells are divided into T1 (CD10^+^CD21^lo^CD27^-^) and T2/3 (CD10^+^CD21^hi^CD27^−^) B cells, while the mature naive B cells are defined as CD10^-^CD20^hi^CD27^−^ cells. Transitional B cells and mature naive B cells express germline-encoded immunoglobulin (Ig) genes of the IgD and/or IgM isotypes. In contrast, memory B cells, plasmablasts, and plasma cells are antigen-experienced cells that in most cases originate from germinal center reactions. Most antigen-experienced B cells have undergone somatic hypermutation (SHM) and class switch recombination to IgG, IgA, or IgE (1), but non-switched memory B cells also exist (2). Resting memory B cells persist by self-renewal, which proliferate and differentiate into plasma cells upon antigen re-exposure. To maintain the lineage following activation, some daughter cells remain as slowly dividing memory B cells, while others become terminally differentiated antibody-secreting cells (ASCs). Whether this is a stochastic process (3) or mediated by directed asymmetric cell division (4) remains a question of debate. Peripheral ASCs, often referred to as plasmablasts, are short-lived and distinct from the long-lived plasma cells found in bone marrow (BM) or other anatomical niches that support their survival (5, 6).

During late-stage B cell development, immature/transitional B cells exit the BM to enter the circulation where they are subjected to peripheral selection. This is at least in part regulated by B cell-activating factor (BAFF), which is present in limited quantities, thereby setting a competitive threshold for B cell survival (7, 8). The surviving mature naive B cells migrate to secondary lymphoid organs, i.e., the spleen, lymph nodes, and mucosa-associated lymphoid tissue. Upon antigen encounter, extrafollicular plasma cell responses resulting in the production of antibodies that have not undergone SHM may occur. However, most B cell responses against protein antigens are T cell dependent and products of germinal center reactions. Here, antigen-specific B cells undergo hypermutation of the encoded antibody sequences to diversify the antigen-specific repertoire and the resulting B cells interact closely with follicular dendritic cells and follicular helper T (Tfh) cells for selection of high affinity B cell clones. The signals that dictate B cell differentiation into memory B cells or plasma cells in the germinal center reaction are only beginning to be understood (9), including the important roles of Tfh cells (10–12). These processes are of high relevance for vaccine research as both memory B cells and plasma cells are needed for sustained humoral immunity.

## B Cell Dysfunction in HIV-1-Infected Individuals

**Figure 1:**
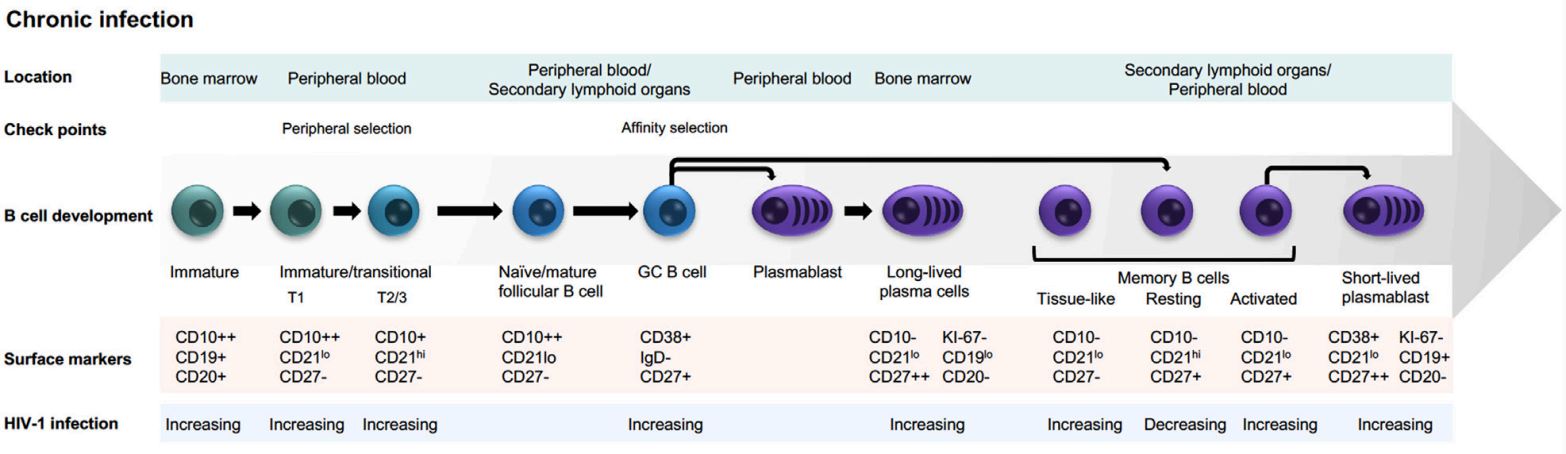
B cell subsets and dysregulation in HIV-1 infection.

During chronic HIV-1 infection, several imbalances in B cell subsets develop (Figure 1), affecting the capacity of chronically infected individuals to respond to vaccination and handle co-infections (13–17). Hypergammaglobulinemia and loss of B cell memory are hallmarks of these humoral immunity alterations (18, 19). Dysregulation of B cells is apparent relatively early after HIV-1 infection and worsens during disease progression. Early introduction of antiretroviral therapy to dampen active viremia has positive effects on preserving B cell subsets (20). Dysregulated B cell subsets and functions are also observed in individuals repeatedly exposed to malaria (19). Thus, B cell alterations in both HIV-1- and malaria-infected subjects are likely consequences of prolonged inflammatory responses that occur under these conditions, rather than caused by direct pathogen–B cell interactions. The specific B cell alterations described in chronically HIV-1-infected individuals include effects on both antigen-inexperienced cells and antigen-experienced cells as discussed below.

### Antigen-Inexperienced Cells

HIV-1-infected individuals display increased frequencies of circulating immature transitional B cells (21). As transitional B cells display increased sensitivity to spontaneous apoptosis, this may lead to a decreased pool of mature naive B cells (22, 23). Altered migratory capacity of immature transitional B cells was also observed, which could affect the distribution of these cells between blood and secondary lymphoid organs in HIV-1-infected individuals (24). Furthermore, as mentioned earlier, peripheral B cell selection is regulated by BAFF, a B cell growth factor shown to be elevated in both chronic infection and autoimmunity (25, 26). BAFF is regulated by type I interferons (27); thus, increased BAFF levels in HIV-1 infection may result from sustained type I interferon responses due to chronic viremia. A potential consequence of increased BAFF levels is that B cell selection thresholds are altered, which may promote survival of B cells that otherwise would be subject to negative selection such as poly-reactive or auto-reactive clonotypes (28, 29). Whether the naive B cell repertoire in HIV-1-infected individuals more frequently display features associated with poly- or self-reactivity is not known but will be important to investigate, especially in relation to the generation of broadly neutralizing antibodies (bNAbs) (30).

### Antigen-Experienced Cells

HIV-1-infected individuals also display alterations of the memory B cell compartment. Activated human memory B cells, defined as CD20^+^/CD21^lo^/CD27^+^, and tissue-like memory B cells, defined as CD20^+^/CD21^lo^/CD27^−^, are increased during persistent HIV-1 infection, whereas resting memory B cells, defined as CD20^+^/CD21^hi^/CD27^+^, are decreased in frequency (13, 31). Consequences of these B cell compartment alterations are observed already early in infection in the form of poor maintenance of serological antibody responses to previous vaccination (i.e., measles, tetanus, and pneumococcus) (17), as well as impaired responses to new vaccinations (32). During the chronic phase of the infection, exhausted B cells also appear. Exhausted B cells are characterized by a decreased capacity to proliferate in response to stimulation (33). The exhausted memory B cell phenotype is reminiscent of that of exhausted T cells with expression of molecules that negatively regulate antigen receptor signaling or homing to sites of inflammation (34, 35). Furthermore, HIV-1-infected individuals display increased frequencies of circulating CD20^−/lo^/CD27^hi^/CD38^hi^ plasmablasts (36) consistent with non-antigen-specific differentiation of memory B cells into ASCs resulting in hypergammaglobulinemia and decreased numbers of resting memory B cells. Thus, the immune system in chronically HIV-1-infected individuals is different from that of healthy subjects in several ways, which likely affects the kinds of antibodies that are elicited. Below, we compare and contrast what is known about the induction of neutralizing antibody responses in chronic infection versus in immunization for an improved appreciation of these differences.

## The Env Trimer as a Neutralizing Antibody Target

The envelope glycoproteins of HIV-1 (Env) are the only virus-encoded antigens exposed on the external surface of the virus particle and thus the sole targets for neutralizing antibodies. The HIV-1 Env spike is composed of a trimer of dimers in a tightly packed infectious entry unit where the external glycoprotein gp120 is non-covalently attached to the transmembrane protein gp41 (37, 38). The native HIV-1 Env trimer complex is meta-stable and readily acquires lower energy forms that are highly immunogenic [reviewed in Ref. (39)]. Antibodies elicited by these non-native forms of Env are non-neutralizing, or only capable of neutralizing sensitive (tier 1) viruses, which are distinctly different from circulating neutralization-resistant (tier 2) virus variants (38).

The functional Env spike is exceptionally well shielded from immune recognition by N-linked glycans that cover most of the Env protein surface (40). The sites for N-linked glycosylation in the primary Env amino acid sequence vary between different virus strains and between different time points of viral evolution of a given strain demonstrating the plasticity of Env. HIV-1 evolves constantly in response to host antibody responses in each chronically infected individual, and neutralization-sensitive viruses are readily eliminated *in vivo* leaving only resistant variants in the circulating pool (41). An interesting recent study demonstrated that currently circulating HIV-1 variants are more neutralization resistant than variants isolated from the beginning of the epidemic, in part due to the acquisition of a denser Env glycan shield over time (42). The intrinsic neutralization resistance of HIV-1 is a major challenge for vaccine development where the goal is to induce antibodies capable of neutralizing a broad range of tier 2 isolates to curb HIV-1 transmissions worldwide.

### Neutralizing Antibodies Elicited by Chronic Infection

**Figure 2:**
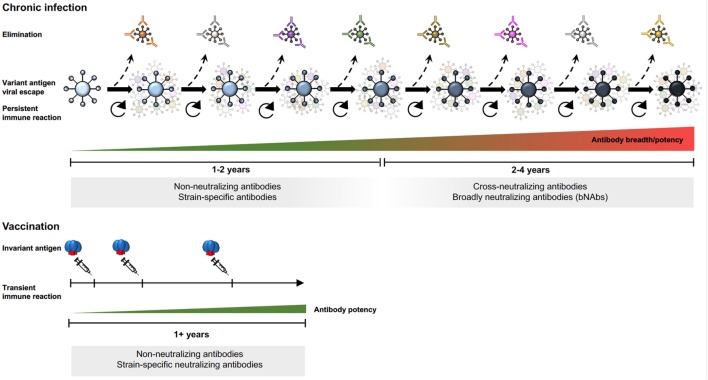
Env-specific antibody responses in chronic HIV-1 infection and after subunit Env vaccination.

Env-specific antibodies generated during the first months of HIV-1 infection are non-neutralizing or strain-specific neutralizing. Non-neutralizing antibodies are elicited by highly immunogenic non-functional forms of Env as mentioned earlier. Strain-specific antibodies neutralize the autologous virus that elicited them but not contemporary viruses that arose subsequently as a result of immune escape from the first wave of antibodies (41). About 2–4 years after the acute of infection, approximately 20% of infected individuals develop cross-neutralizing antibodies (Figure 2) and 1–2% of infected individuals develop bNAbs, which exhibit exceptionally potent neutralizing capacity against a large proportion of virus isolates (43, 44). Isolation and mapping of bNAbs at the monoclonal antibody level allows definition of their target epitopes, revealing sites of vulnerability on the virus that can be targeted by epitope-focused vaccine approaches (45–52).

Since chronic HIV-1 infection is characterized by an arms race between viral evolution and the adaptive immune response, new epitopes are continuously generated, sequentially driving the B cell repertoire toward the generation of bNAbs (53–55). The extensive antigenic variability in Env results mainly from the error-prone HIV-1 reverse transcriptase, which generates swarms of variants in each infectious cycle from which immune escape variants are selected. Despite the high antigenic variability of HIV-1 Env, some determinants are conserved as mutations in these elements compromise viral fitness. These regions are targets for bNAbs and include the primary receptor binding site, the CD4bs, certain variable region 2 (V2) determinants in the trimer apex, the base of the V3 region, and the gp120–gp41 interface region [reviewed in Ref. (56)]. In the case of bNAbs targeting the V3 base, the surrounding N-glycans are often part of the epitope (57, 58). The glycan reactivity observed in many HIV-1-infected individuals (59) is intriguing since antibodies against N-linked glycans is essentially a response against self-structures, which is uncommon in healthy subjects. Thus, the development of such antibodies in chronic HIV-1 infection may reflect a relaxation of peripheral check-points allowing potentially self-reactive B cells to escape negative selection (60).

Several studies have shown that bNAbs possess a high degree of divergence from their corresponding germline antibody sequences, indicating extensive SHM of the antibody sequences (57, 58, 61). High SHM suggests that multiple rounds of affinity maturation and selection in germinal centers have occurred, which appears to be required to develop features associated with broad HIV-1 neutralization. High levels of SHM are not unique to bNAbs but are generally seen in HIV-1 infection (62), as well as in other chronic infections and some settings of autoimmunity (63). This suggests that extensive SHM is a consequence of prolonged antigen exposure and persistent inflammatory responses, processes that allow selection of B cells over long periods of time. However, it is likely that not all changes introduced by SHM are required for bNAb activity as shown for the bNAb VRC01, where a subset of the amino acid changes that differed between the mature antibody and the assigned germline VH1-2*02 sequence were sufficient to confer bNAb activity (64).

The high degree of divergence of bNAb sequences from their germline Ig gene segments complicates the process of inferring the unmutated recombined ancestor sequences for these antibodies. Studies of germline-reverted bNAb sequences have shown that they rarely bind Env suggesting that they possess very low initial affinities to the unmutated BCR (65). However, in most cases where this was studied, the Env present in the patient at the time of elicitation of the bNAb lineage was not known. An exception to this is the identification of antibody CH103, which binds the presumed transmitted/founder Env in its germline-reverted form (55). The lack of Env binding to germline-reverted bNAbs may be explained by the fact that some human germline variable (V) alleles are missing in the current databases, which could affect the processes of germline reversion (66). In support of this, it is becoming increasingly clear that there are more human antibody V alleles than previously appreciated (67–70). An improved understanding of human antibody germline genes is therefore needed. We recently reported that next-generation sequencing (NGS) coupled with a new computational tool, IgDiscover, can accelerate the definition of germline-encoded Ig gene segments and allow higher-throughput studies (70).

HIV-1 bNAb sequences stand out not only because of high levels of divergence from their germline sequences in terms of single nucleotide differences but also because they frequently display insertions and deletions (indels) introduced during the process of SHM (71). Indels, which are rarely seen in antibodies elicited in healthy subjects, generate further diversity in infection-induced Env-specific antibody repertoires, an area that is only beginning to be understood. The present increase in NGS-based antibody repertoire analysis provides highly valuable information about how the human B cell response evolves during chronic infections. Another characteristic feature of some classes of HIV-1 bNAbs, such as the apex-targeting antibodies, is their exceptionally long heavy chain complementarity-determining region 3 sequences. B cells encoding BCRs with such long HCDRs are rare in the naive B cell population but appear to be preferentially selected in Env-specific responses, at least in a subset of individuals. This feature is likely required for the antibodies to penetrate the dense glycan shield and bind conserved determinants at the Env trimer apex (72, 73). Collectively, these genetic features demonstrate that HIV-1 antibodies are highly selected and bNAb specificities arise from extensive co-evolution processes between the virus and responding B cells.

### Neutralizing Antibodies Elicited by Subunit Env Vaccination

The persistent B cell selection observed during chronic HIV-1 infection is in stark contrast to the transient response that takes place following vaccination with non-replicating subunit vaccines. Highly mutated antibodies are not induced by current immunization regimens but might be achievable by using heterologous Env immunogens administered in a sequential manner to promote responses to common determinants on HIV-1 Env. So far, bNAbs have not been elicited by immunization of primates with natural immune repertoires. Given that bNAb development in infection depends on extensive B cell selection on a constantly changing pool of virus escape variants, it is not surprising that conventional immunization regimens do not induce bNAb specificities. It is also not known if certain precursor populations are lost during peripheral B cell selection processes, which are known to be under tighter control in healthy vaccine recipients than in chronically infected HIV-1 individuals as mentioned earlier.

Immunization studies using early generation Env trimers provided valuable information about the B cell response elicited in both small animals and in primates. While tier 1-neutralizing antibody responses are readily induced, tier 2-neutralizing responses are mostly limited to autologous tier 2 responses (Figure 2) (74, 75). For a detailed understanding of epitope-specific antibody responses induced by vaccination, methods for antibody specificity mapping and isolation of monoclonal antibodies are needed. Such methodologies are under continuous development to facilitate analyses of vaccine-induced responses at a higher level of resolution [reviewed in (76)]. Results from immunized non-human primates demonstrate that Env vaccine-induced responses consist of many different clonotypes, most of which appear to be modestly expanded (77–79). Highly polyclonal B cell responses are also observed in humans vaccinated with tetanus toxoid, another protein subunit-based vaccine, administered using a homologous prime-boost regimen (80, 81). It is perhaps not surprising that vaccine regimens based on homologous boosting result in polyclonal B cell responses with modest levels of SHM where each clonotype has reached an affinity ceiling to the invariant vaccine antigen (82), rather than being driven by a constantly changing antigen that repeatedly resets the affinity threshold for B cell selection, as is the case in HIV-1 infection.

Despite the many contrasts between chronic infection and vaccination, dissection of Env vaccine-induced antibody responses at the monoclonal level has also revealed similarities in terms of the targeted epitopes. For example, antibodies against non-neutralizing epitopes in gp41 as well as against tier 1-neutralizing epitopes in variable region 3 (V3) are readily elicited in both settings suggesting that these specificities are abundant in the naive B cell repertoire in both humans and commonly used animal models as shown by monoclonal antibody isolation (78, 83, 84). Similarly, CD4bs-directed antibodies capable of neutralizing tier 1 viruses, exemplified by the non-broad neutralizing antibody F105, are elicited both in infection (85) and in vaccination of non-human primates (86). The availability of protocols for efficient cloning of antibodies from non-human primates (86, 87) has facilitated such studies and are now widely used to dissect vaccine-induced responses in rhesus macaques. With the exception of one study (88), less is known about epitope-specific antibody responses in immunized rabbits where germline Ig genes so far are insufficiently characterized, currently hampering monoclonal antibody isolation in this model.

While early generation HIV-1 Env vaccine candidates were poor mimics of the functional Env spike, recent work has resulted in immunogens that better mimic the native viral spike. The definition of a native spike structure is that bNAbs epitopes are retained while non-neutralizing Ab epitopes are not. Soluble trimeric Env immunogens that meet these criteria include the BG505 SOSIP trimers and the Native Flexibly Linked (NFL trimers) (89–91) for which high-resolution structures were obtained (92–94). Emerging *in vivo* evaluation of the immunogenicity of these trimers, when used in homologous prime-boost regimens, demonstrates that they elicit autologous tier 2-neutralizing antibody responses but limited neutralization breadth (95). The epitopes mediating strain-specific neutralization may be different for different HIV-1 strains, or in different host species, as exemplified by the finding that antibodies against the V2 region mediate the autologous neutralizing activity induced by clade C 16055 trimers in NHPs (95), while antibodies against the gp120-gp41 interface mediate the autologous neutralizing activity induced by clade A BG505 trimers in rabbits (88). The role played by potential differences in host B cell repertoires in terms of the specificities induced by a given immunogen remains insufficiently understood but will be important to determine to better understand predictability of different animal models for assessment of human vaccine candidates. In this respect, it was shown the same immunogen that elicits potent autologous neutralizing antibodies in rabbits fails to do so in mice (96). Further work is required to define similarities and differences in germline antibody genes and expressed repertoires between commonly used animal models, including small animals, NHPs, and humans.

In conclusion, while much has been learnt from studying the development of bNAbs in chronic HIV-1 infection, focused efforts are now needed to translate these findings to the setting of vaccination. Given the challenge of this goal, achieving this will require coordinated vaccine evaluation trials in both well-chosen animal models and in humans.

## Author Contributions

MS created the figures. MS and GKH jointly wrote the manuscript.

## Conflict of Interest Statement

The authors declare that the research was conducted in the absence of any commercial or financial relationships that could be construed as a potential conflict of interest.
